# Interval uncertainty analysis of a confined aquifer

**DOI:** 10.1038/s41598-021-86118-0

**Published:** 2021-03-22

**Authors:** Chengcheng Xu, Chuiyu Lu, Jianhua Wang

**Affiliations:** 1 School of Earth and Environment, Anhui University of Science and Technology, Huainan, 232001 China; 2 State Key Laboratory of Water Cycle Simulation and Regulation, China Institute of Water Resources and Hydropower Research, Beijing, 100038 China

**Keywords:** Environmental sciences, Hydrology

## Abstract

Water inflow forecast is influenced by many factors and yields uncertain results. To more accurately predict the magnitude of water inflow and quantitatively define the corresponding response in the parameter change interval, this study combined a non-probabilistic set theory and uncertainty analysis to derive an equation for the confined water inflow. Using mining area data and comparing the calculation of upper and lower boundary limits obtained by a Monte Carlo method, results of the confined water inflow equation were calculated with relative errors of 5% and 10%. When corresponding to the rate of change of the variable parameter, the results showed that under the same error conditions, the allowable rate of change when calculating the minimum value using Eq. A was greater than when using Eq. B, and the maximum value using Eq. B yielded a greater allowable rate of change than the maximum value calculated by Eq. A. Thus, the obtained rate of change for Eq. A is indicative of the lower limit, and Eq. B is conducive to the calculation of the upper limit of mine water inflow.

## Introduction

Water damage is a key problem during mining^1–3^. The large-well method is commonly used for predicting mine water inflow^4–6^, however, the accuracy of the results are subject to the constraints of hydrogeological conditions^7–9^. For example, the calculation process of the large well method is simple, but for areas with complex hydrogeological conditions, the calculation accuracy needs to be further improved. Specifically, the current problem of water inrush in day mining puts great significance on rapidly diminishing the uncertainty of water inflow prediction^10,11^, and the complexity of hydrogeological conditions in mining areas has become one of the key issues of groundwater science^12–14^. In the study of uncertainty, randomness, gray, and fuzzy mathematics, among others, are commonly used methods^15,16^. Owing to the incompleteness of the field data, it is difficult to obtain probability density functions using random mathematical methods, resulting in large errors in the calculation of water inflow that are prohibitive mine exploitation^17,18^. The use of deterministic, large-well method calculations similarly do not rule out the effects of changes in hydrogeological conditions. Starting from the uncertain boundary, and combining with the non-probability set theory convex model method^19–21^, the equation for calculating the water inflow with upper and lower limits can be derived. From the initial deterministic, simple large-well method, to the consideration of uncertain parameter variation fused with the large-well method, the uncertain factors in the calculation can be expressed in the form of interval change, and integrated into the calculation^22^. Thus, this calculation of mine water inflow provides a new way to predict water inflow in complex conditions, and represents a significant advancement for mining.

## Prediction equation for interval water inflow

The equation of confined water is the most commonly used mathematical equation for calculating mine water inflow, and defined using the following equations^23^:

1 $$ Q = 2.73\frac{KMS}{{\lg \left( {\frac{{R_{0} }}{{r_{0} }}} \right)}} $$

2 $$ R = 10S\sqrt K $$

3 $$ r_{o} = \eta \left( {\frac{a + b}{4}} \right) $$

4 $$ R_{0} = r_{0} + R $$

where Q is water inflow (m^3^·day^−1^); K is hydraulic conductivity (m·day^−1^); a and b are working face length and width, respectively (m); $\eta$ is the calculation factor (see Table 1); r_0_ is the reference radius (m); R is the influence radius (m); R_0_ is the large well reference radius (m); M is the thickness of the aquifer (m); and, S is the drawdown of the water table (m).

**Table 1:** Relationship of b/a and $\eta$.

b/a	0	0.20	0.40	0.60	0.8	1.00
$\eta$	1.00	1.12	1.14	1.16	1.18	1.18

The assumptions according to Eq. (4) are that the aquifer is almost horizontal, the distance between the top and bottom plates is relatively uniform, the water medium is relatively uniform, thus avoiding the fluctuation of water flow in the aquifer, and there is a certain range in the pumping well with a circular long radius head boundary. However, during the actual mining process, owing to the possible existence of faults, geological anomalies, and other extraneous factors, the formation permeability coefficient of the study area is locally variable; thus, the aquifer is characterized by non-uniformity, and it is difficult to achieve a circular or rule head boundary.

In the current prediction of mine water inflow, with a series of measurements such as mining area drainage, the groundwater level changes through a gradual process. Hence, the groundwater flow is similar to the steady flow, and the heterogeneous aquifer is regarded as stable. The flow is calculated using the traditional large-well method. Equation (1) is calculated under the assumption that the calculation object is approximated to a steady flow, and Q is nonlinear with K, M, S, and r_0_. The rate of change of the five variables K, M, R, S, and r_0_ represents the variation interval of the variable Table 2. It is convenient to use Eq. (1) to calculate water inflow after considering the influences of the parameter changes.

**Table 2:** Interval equations of water inflow.

Empirical equation (Eq. A)	${\mathrm{Q}}_{0}\pm \frac{2}{B}\sqrt{{{\mathrm{Q}}_{0}}^{2}{(\mathrm{Y}-0.5\mathrm{X})}^{2}{{\upbeta }_{\mathrm{K}}}^{2}+{{\mathrm{Q}}_{0}}^{2}{\mathrm{X}}^{2}{{\upbeta }_{{\mathrm{r}}_{0}}}^{2}+{{\mathrm{Q}}_{0}}^{2}{\mathrm{Y}}^{2}{{\upbeta }_{\mathrm{M}}}^{2}+{(2.73\mathrm{KMS}-{\mathrm{Q}}_{0}\mathrm{X})}^{2}{{\upbeta }_{\mathrm{S}}}^{2}}$
Actual survey (Eq. B)	${\mathrm{Q}}_{0}\pm \frac{\sqrt{5}}{B}\sqrt{7.45{\mathrm{K}}^{2}{\mathrm{M}}^{2}{\mathrm{S}}^{2}{{\upbeta }_{\mathrm{M}}}^{2}+{{\mathrm{Q}}_{0}}^{2}{[\mathrm{Y}}^{2}{{\upbeta }_{K}}^{2}+{\mathrm{X}}^{2}{{\left({{\upbeta }_{\mathrm{R}}}^{2}+{{\upbeta }_{{r}_{0}}}^{2}\right)]}}}$

Q_0_ is the result when the corresponding variable takes the center of the interval. ${\upbeta }_{\mathrm{K}}$= $\Delta$ K/$\Delta$ K_0_, ${\upbeta }_{\mathrm{M}}$= $\Delta$ M/$\Delta$ M_0_,${\upbeta }_{{r}_{0}}$= $\Delta$ r/$\Delta$ r_0,_${\upbeta }_{\mathrm{R}}$= $\Delta$ R/$\Delta$ R_0_,${\upbeta }_{\mathrm{S}}\hspace{0.17em}$= $\Delta$S/$\Delta$S_0_, ${\upbeta }_{\mathrm{K}}$, ${\upbeta }_{\mathrm{M}}$, ${\upbeta }_{{r}_{0}}$, ${\upbeta }_{\mathrm{R}}$, and ${\upbeta }_{\mathrm{S}}$ are the rates of change of the corresponding variables. The “+” in “±” corresponds to the upper limit (maximum value) of the water influx change interval, and the “−” corresponds to the lower limit (minimum value).

## Accuracy of the interval water forecasting equation

Based on a first-order Taylor series and the optimization theory, the obtained rate of change of the equation in Table 2 must be finite. The actual upper and lower limits of the response interval obtained by the Monte Carlo method were used to analyze the validity of the equation and the rate of change limit in Table 2, and the results are shown in Table 3.

**Table. 3:** Calculation rate of water inflow under different error conditions.

Equation	Error boundary	Parameter	Data 1	Data 2	Data 3	Data 4	Data 5
		Q_0_ (m^3^ day^−1^)	1200	2400	4000	7000	9000
		M_0_ (m)	40	80	120	150	180
		S (m)	10	25	40	60	80
		K (m day^−1^)	0.0005	0.005	0.008	0.01	0.05
A	The allowable rate when the absolute value of the maximum relative error is less than β	β = 5%	0.109	0.114	0.158	0.167	0.18
		β = 10%	0.146	0.237	0.257	0.266	0.304
	The allowable rate when the absolute value of the minimum relative error is less than β	β = 5%	0.165	0.172	0.257	0.281	0.319
		β = 10%	0.174	0.178	0.292	0.35	0.338
B	The allowable rate when the absolute value of the maximum relative error is less than β	β = 5%	0.131	0.198	0.226	0.268	0.297
		β = 10%	0.274	0.285	0.355	0.366	0.392
	The allowable rate when the absolute value of the minimum relative error is less than β	β = 5%	0.162	0.193	0.21	0.231	0.275
		β = 10%	0.178	0.199	0.276	0.278	0.343

The maximum relative error in “the allowable rate of change of the absolute value of the maximum relative error is smaller than the value of the variable” is the error between the upper limit value Q^+^ of the water inflow according to the equation in Table 2, and the value derived using the Monte Carlo method^24^. In the calculation process shown in Table 2, the rate of change ranges from 0 to 0.5, with increments of 0.01.

For the calculation of Eq. B for each set of data, the influence radius value (R) calculated by Eq. (2) was multiplied by 4, the water inflow amount was calculated using Eq. (1), and the other parameters were held constant. Table 3 shows the maximum rate of change of the corresponding variables of the five sets of test data for the two interval water inflow prediction equations at error levels β = 5% and 10%. Data 1, for example, used Eq. (1) to calculate the water inflow, and if the absolute value of the relative error of the calculated maximum value did not exceed 0.05, then the rate of change of the four parameters in Eq. (1) could not exceed 0.11. If the absolute value did not exceed 0.1, then the rate of change of the four parameters could not exceed 0.15.

It can be seen from Table 3 that under the same error requirement, the upper limit is greater than the lower limit when using Eqs. A and B. That is, when the rate of change of the variable is relatively large, the reliability of the calculated maximum water inflow using Eq. (1) is higher than the minimum water inflow. When it is necessary to obtain the upper and lower limits of the water inflow within the larger change interval and meet certain accuracy requirements, the interval can be divided into cells, and then the Eqs. A and B in Table 2 can be used between the cells.

## Application

The aquifer in the Jurassic era of one mine was primarily composed of coarse sandstone. The average elevation of the aquifer was 838.18 m. According to the drilling data of the working face, the average thickness of the Jurassic-era system was 108 m. For the mining area, some boreholes were laid and pumping tests were carried out, and the hydrogeological parameters of the area were obtained. The aquifer had a maximum permeability coefficient of 0.0654 m day^−1^, a minimum of 0.00043 m day^−1^, an average of 0.02265 m·day^−1^, a single-hole water inflow of 0.05–3.85 L s^−1^, and a unit water inflow of 0.0015–0.1171 L s^−1^ m^−1^. The layer was water-invariant, an indirect water-filled aquifer mined by the 3–1 coal seam, and was also the main aquifer. There were 14 normal faults, one reverse fault, one fault with a drop of more than 10 m, two fault gaps from 5 to 10 m, two fault gaps from 3 to 5 m, and the remaining nine faults were < 3 m. After the working face of the mine was drained, the pressure outside of the funnel boundary was confined water, while inside of the funnel was unconfined water. The drawdown of the groundwater table was 351.45 m, equaling the water floor level (838.18 m) subtracted from the confined water level (1189.63 m). Equation A was used to calculate the corresponding water influx change interval under each variable interval. Considering the incompleteness of the existing mine data and the uncontrollability of the actual hydrogeological conditions, $\upbeta$_s_ = 0. 05, $\upbeta$_M_ = $\upbeta$k = $\upbeta$r_0_ = 0.2; that is, the variation range of the water level depth (m) was [7.64, 84.29], the aquifer thickness (m) was [38.3, 188.04], the permeability coefficient was [0.00043, 0.0654], and the equivalent radius (m) was [972.67, 2059.4]. After calculation, the variation range of water inflow (m^3^ day^−1^) was [9.48, 5127.56]. There was a bounded difference in the amount of water inflow calculated using the maximum, minimum, and average values of variables, indicating that the variable interval has practical significance for the prediction of water inflow.

**Figure 1:**
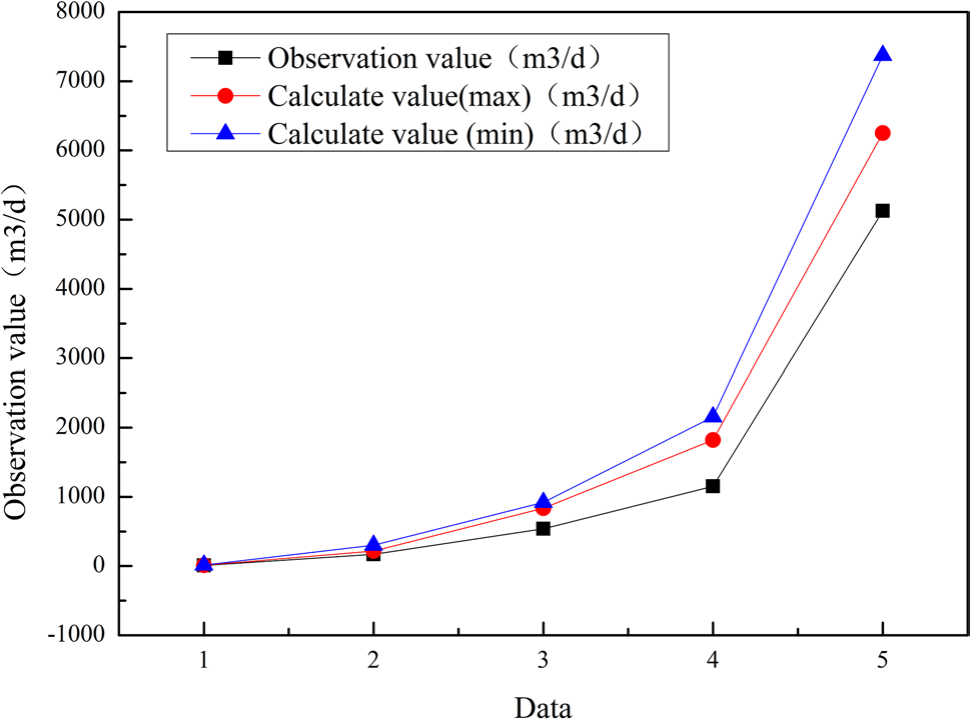
Comparison of calculated and observed values.

In order to further explore the relationship between the calculated results of Eq. A and the rate of change of each variable, a comparison analysis was performed Fig. 1. Analyzing the data, the value calculated by the formula is larger than the observed value. The calculated maximum value is closer to the observed value than the minimum value. Thus proved the reliability of the calculated maximum water inflow using Eq. (1) is higher than the minimum water inflow.

**Figure 2:**
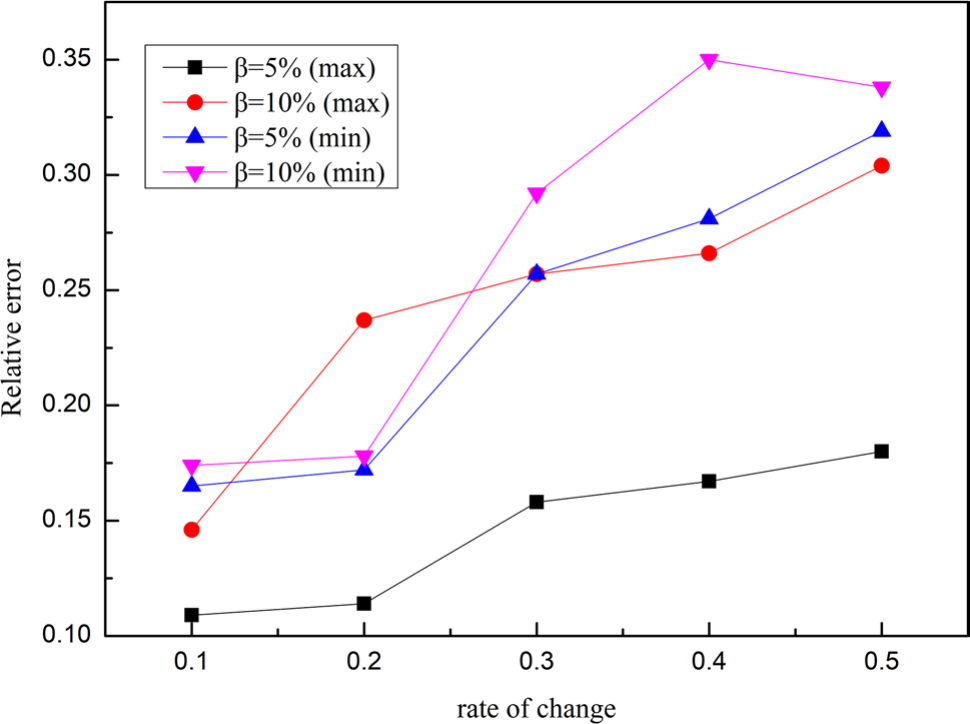
Relative error with the corresponding rate of change.

In Fig. 2, when the rate of change of the variable was 0.3, the error of the maximum value was substantially < 20%. Further, when the rate of change was 0.2, the minimum value error was substantially < 10%. The actual calculated values revealed that the interval water inflow calculated by the empirical equation was adaptive within a certain range of variables. It can be concluded from Fig. 2 that there is a nonlinear relationship between the relative error, which is calculated by the empirical equation and the rate of change of the variable.

## Discussion and conclusion

### Discussion

Most of the existing theories and methods for studying uncertain structures assume that the uncertainty of the structure is a random variable or a random process, and satisfies a certain probability distribution assumption. In this case, the rationality of structural analysis and design can be justified; however, the probability density of uncertain variables and experimental information about the structure are often lacking. If these probability distribution assumptions are not satisfied, the rationality of structural analysis and design lose much of their value. It is generally difficult to verify whether the variables of the actual structure satisfy a certain assumption, and this results in the following contradiction: Although the complexity of the actual structure is acknowledged, it is not always possible to use simple model systems; but alternatively, the assumptions of the analysis model due to the uncertain variables are artificial, so almost all uncertain variables can obtain estimates that are arbitrarily close to the real system through probability models. It is precisely because of this contradiction that the use of non-probabilistic models to study various uncertainties is gaining popularity. The equations proposed in this study take into account the uncertainty of the interval and is more applicable to areas with complicated hydrogeological conditions. When calculating the maximum water inflow in a mining area, the formula given in this study is more accurate under the same parameters.

### Conclusion

1. An uncertainty analysis of the interval, combined with the theory of a non-probability set, allowed the prediction equation of confined water inflow to be derived. This equation considered the traditional large-well method, and as the parameters changed, the amount of water influx also changed within a certain interval.
2. The mathematical equation derivation and the calculation of the mining area example revealed that the predictive accuracy of the equation for the interval water inflow could be improved for magnitude, and provide some reference for the calculation of water inflow in mining areas.

## Supplementary information

Supplementary information.
