# Supplementary material for: Interval uncertainty analysis of a confined aquifer
Source: Sci Rep. 2021 Mar 22;11:6516. doi: 10.1038/s41598-021-86118-0 (PMC7985385; doi:10.1038/s41598-021-86118-0)
Supplement: Supplementary file 1 — Supplementary information. [file 41598_2021_86118_MOESM1_ESM.docx]

**Interval uncertainty analysis of a confined aquifer**

*Chengcheng Xu ^1^, Jianhua Wang ^2^, Chuiyu Lu ^2^*

*1. School of Earth and Environment, Anhui University of Science and Technology; Huainan, China 232001;*

*2. State Key Laboratory of Water Cycle Simulation and Regulation, China Institute of Water Resources and Hydropower Research; Beijing, China 100038*

**Annex**

**Non-probability set theory convex model method**

Let β^0^ = (βi^0^)n = (β_1_^0^,β_2_0,...,β_n_0 )^T^ be the statistical mean or center number of the parameter β = (β_i_)n = (β_1_,β_2_,...,β_n_)^T^ of the analytical model. The parameter can be expressed as

β =β^0^+ε (Eq. A1)

According to the set theory convex model, the statistical mean of the function in the bounded uncertain parameter β =(β_i_)n = (β_1_，β_2_ ，…，β_n_)^T^ is β^0^ = (β_i_^0^)n = (β_1_^0^，β_2_^0^ ，…，β_n_^0^ )^T^, is carried out near Taylor and retains one item, yielding:

φ(β) = φ(β^0^ +ε) = φ(β^0^ ) + $\sum_{i=1}^{n} \frac{\partial\varphi(\beta0)}{\partial(\beta i)}\varepsilon i$= φ_0_ + f^T^ε (Eq. A2)

It is assumed that the uncertainty ε of the bounded uncertain parameter varies near the statistical mean within the bounded convex set of equation (Eq. A3), namely:

C(ε，δ) = {ε: ε^T^Mε ≤ δ^2^ } (Eq. A3)

where M is the positive definite matrix, and δ is the positive real number.

When the bounded uncertain parameter ε = (ε_i_ ) _n_ = (ε_1_，ε_2_，…，ε _n_ )^T^ varies within the bounded convex set of Eq. A3, the upper and lower limits of the bounded uncertain response of the function can be determined by Taylor's first-order approximate solution φ = φ max, and φ = φ min, respectively:

φ = φ max = max_δ∈E(ε，δ)_{φ_0_ + f^T^ε} (Eq. A4)

φ = φ min = min_δ∈E(ε，δ)_{φ_0_ + f^T^ε} (Eq. A5)

Mathematical optimization theory has confirmed that the extreme values of Eqs. A4 and A5 will be reached at the boundary of the ellipsoid region represented by Eq. A3. Thus, the Lagrangian function will be:

L = φ_0_ + g^T^ε + w(ε^T^ Mε-δ^2^ ) (Eq. A6)

where w is the Lagrange multiplier. The necessary conditions for taking the extreme value are defined in Eq. A7:

$\frac{\partial L}{\partial\varepsilon}$= f + 2wMε = 0 (Eq. A7)

Thus:

w^2^ = $\frac{1}{4\delta^{2}}$ f ^T^ M^-1^ f (Eq. A8)

w=± $\frac{1}{2\delta}\sqrt{f^{T}M^{-1}f}$ (Eq. A9)

Substituting Eq. A9 into Eqs. 4 and 5 yields:

φ = φ_max_ = φ_0_ + δ$\sqrt{\sum_{j=1}^{n} {(\frac{n}{2}\Delta\beta j\frac{\partial\varphi\beta^{0}}{\partial\beta j})}^{2}}$ (Eq. A10)

φ = φ_min_ = φ_0_ - δ$\sqrt{\sum_{j=1}^{n} {(\frac{n}{2}\Delta\beta j\frac{\partial\varphi\beta^{0}}{\partial\beta j})}^{2}}$ (Eq. A11)
